# Neglected parasitic diseases from a one-health perspective: American trypanosomiasis and leishmaniasis in dogs and humans in the Bolivian Chaco

**DOI:** 10.1186/s13071-025-07044-y

**Published:** 2025-11-27

**Authors:** Fabio Macchioni, Ettore Napoli, Habimael Castrillo Tarraga, Giovanni De Benedetto, Esther Tapia Vega, Adriana Augello, William Medina Ustarez, Maribel Mendoza Moreno, Patricia Rojas Gonzales, Francesco Cosmi, Emanuele Brianti, Simona Gabrielli

**Affiliations:** 1 Department of Veterinary Sciences, University of Pisa, Pisa, Italy; 2 Department of Veterinary Sciences, University of Messina, Messina, Italy; 3 Zoonosis Unit, Villa Montes, Bolivia; 4 Department of Public Health and Infectious Diseases, Sapienza University, Rome, Italy; 5 Facultad Integral del Chaco, Universidad Autónoma Gabriel René Moreno, Camiri, Bolivia; 6 Cordillera Health District, Santa Cruz Department, Camiri, Bolivia; 7 Convenio Ministerio de Salud, Vicariato de Camiri, Camiri, Bolivia

**Keywords:** *Leishmania*, *Trypanosoma cruzi*, Bolivia, qPCR, Rapid Diagnostic Test, ELISA, Dogs

## Abstract

**Background:**

This study investigates the prevalence of leishmaniasis and American trypanosomiasis (Chagas disease) – two neglected vector-borne diseases – in humans and dogs in the Bolivian Chaco region, where high poverty levels increase population vulnerability. Leishmaniasis, which affects millions globally, is widespread in Bolivia, a country reporting some of the highest rates of cutaneous and mucocutaneous cases in Latin America. Chagas disease is endemic across the region, with an estimated 4.5 million individuals affected.

**Methods:**

Blood and serum samples were collected from 189 dogs living in rural communities near the cities of Camiri and Villa Montes. Samples were tested using serological assays and quantitative PCR (qPCR) to detect *Leishmania* spp. and *Trypanosoma cruzi*. Additionally, serum samples from 151 school-aged children from both areas were screened for anti-*Leishmania* antibodies.

**Results:**

The seroprevalence of *Leishmania* in dogs was significantly higher in Villa Montes (46%) compared with Camiri (26%). Moreover, *Leishmania* DNA was detected by qPCR in 9 out of 125 dogs tested (7.2%). Among children, 13.2% from Villa Montes tested positive for *Leishmania* antibodies, while no positive cases were found in Camiri. Serological evidence of previous *T*. *cruzi* infection was identified in 17.7% of dogs, although all qPCR results for *T*. *cruzi* were negative.

**Conclusions:**

These findings highlight the relevance of a One Health approach, as dogs may serve as reservoirs for both parasites, potentially increasing the risk of human transmission. Integrated control measures – including vector management and ongoing surveillance – are essential to reduce transmission and protect public health. Future research should prioritise mapping infection patterns and exploring ecological factors influencing disease dynamics.

**Graphical Abstract:**

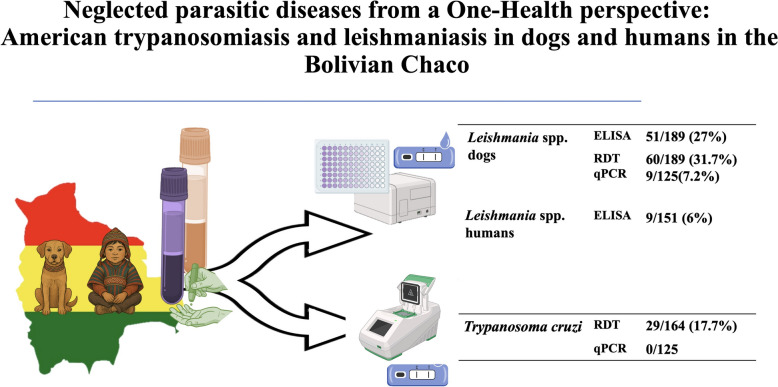

**Supplementary Information:**

The online version contains supplementary material available at 10.1186/s13071-025-07044-y.

## Background

Leishmaniasis and Chagas disease (CD) are neglected tropical diseases caused by closely related kinetoplastid protozoan parasites, both transmitted by insect vectors. These diseases disproportionately affect impoverished populations and have historically received limited investment in research and in the development of innovative preventive strategies [1].

Leishmaniasis encompasses a group of parasitic infections transmitted to mammals, including humans, via the bite of infected female sand flies. Different *Leishmania* species can cause a spectrum of clinical manifestations in humans, ranging from self-limiting cutaneous lesions to life-threatening visceral disease [2]. The clinical outcome depends on a complex interplay between parasite characteristics and the host immune response. Leishmaniasis remains a major global health issue, with over one billion people living in areas at risk of infection. According to the World Health Organization, an estimated 600,000 to 1 million new cases of cutaneous leishmaniasis (CL) and 50,000 to 90,000 cases of visceral leishmaniasis (VL) are reported annually. However, many cases are likely to go unreported [3].

In the Americas, CL and mucocutaneous leishmaniasis (MCL) remain endemic, with 34,954 cases reported in 2023. While Brazil continues to report the highest number of cases, Colombia and Peru also contribute significantly, reflecting a wider regional distribution than in previous years. Visceral leishmaniasis is also a continuing public health concern, with 73,092 cases reported between 2001 and 2023 – an average of over 3000 cases per year – most of which occurred in Brazil [4].

Between 1983 and 2000, the Plurinational State of Bolivia reported the highest incidence of CL and MCL in Latin America, with *Leishmania braziliensis* identified as the primary aetiological agent. During this period, a total of 31,095 CL and 4,619 MCL cases were documented [5]. In 2022, 2,197 new cases were reported in Bolivia, according to the Pan American Health Organization [6]. In contrast, VL – caused by *Leishmania infantum* – has historically been considered rare in Bolivia. Between 1939 and 2018, only 56 cases were reported, primarily in the Yungas subtropical forest region of north-eastern Bolivia [7, 8].

However, this epidemiological pattern appears to be shifting. In 2022, a VL outbreak occurred in the Bolivian Chaco region (southeastern Bolivia), with 29 cases reported, the majority in children. The outbreak was concentrated in the municipalities of Villa Montes, Bermejo and Yacuiba, with Villa Montes alone accounting for 20 of the 29 cases (personal data, Zoonosis Unit, Villa Montes). In 2024, the Bolivian Ministry of Health reported four additional VL cases in Villa Montes and in Roboré, located in the Santa Cruz Department, where previous entomological and canine studies had indicated a high transmission risk [9].

Despite the widespread burden of human leishmaniasis, data on canine leishmaniasis (CanL) in Bolivia remain extremely limited, with only a few outdated reports available [10, 11].

Beside leishmaniasis, Chagas disease represents another major kinetoplastid infection endemic to Latin America. Although transmission is primarily confined to continental Latin America, imported and vertically transmitted cases have also been reported in non-endemic regions, including North America, Europe and Asia [12]. CD is caused by the protozoan *Trypanosoma cruzi*, which is primarily transmitted to humans and other mammals by triatomine bugs (Hemiptera: Reduviidae) [13]. It remains a significant public health concern in endemic areas, affecting approximately 4.5 million people [14]. Of these, an estimated 30–40% are at risk of developing severe complications, including cardiomyopathy, digestive mega syndromes or both [15].

In Bolivia, which spans approximately 600,000 km^2^, around 80% of the territory is considered endemic for *T*. *cruzi*, placing an estimated 1.8 million people at risk of infection [16]. The Gran Chaco region – including the Bolivian Chaco, a semi-arid and sparsely populated area in the country’s southeast – is recognised as one of the world’s major CD hotspots [17]. While the overall seroprevalence in the general population has declined over the past 40 years [18], studies have reported a prevalence of up to 20% among children in rural communities. The sharp increase in prevalence between the ages of 2 and 15 years suggests that vector-borne transmission is the principal route of infection in this age group [19]. Other studies have similarly reported persistent transmission and seroprevalence rates of 22% in school-aged children [20, 21]. Moreover, over 20% of dogs in the Bolivian Chaco have tested seropositive for *T*. *cruzi*, highlighting the potential role of dogs in domestic transmission cycles [22].

This study was conducted as part of a broader surveillance initiative on canine leishmaniasis (CanL), coordinated by the Bolivian Veterinary Authorities (Zoonosis Unit, Villa Montes) since September 2022. The aim was to investigate the circulation of *Leishmania* spp. in owned dogs and children in two selected regions of Bolivia. In addition, canine samples were tested for *T*. *cruzi* to assess the potential role of dogs as reservoir hosts.

## Methods

### Study area

The Bolivian Chaco is a semi-arid and sparsely populated region located in the southeastern part of the Plurinational State of Bolivia, between longitudes 64°30′ and 58°50′ W and latitudes 17°58′ and 22°20′ S. The majority of the population lives in rural communities, where housing typically consists of rudimentary structures made from wattle-and-daub or unplastered adobe bricks, often with thatched roofs. Poor sanitation and a high level of cohabitation between humans and domestic animals – such as dogs, chickens and pigs – are commonly observed.

**Fig. 1:**
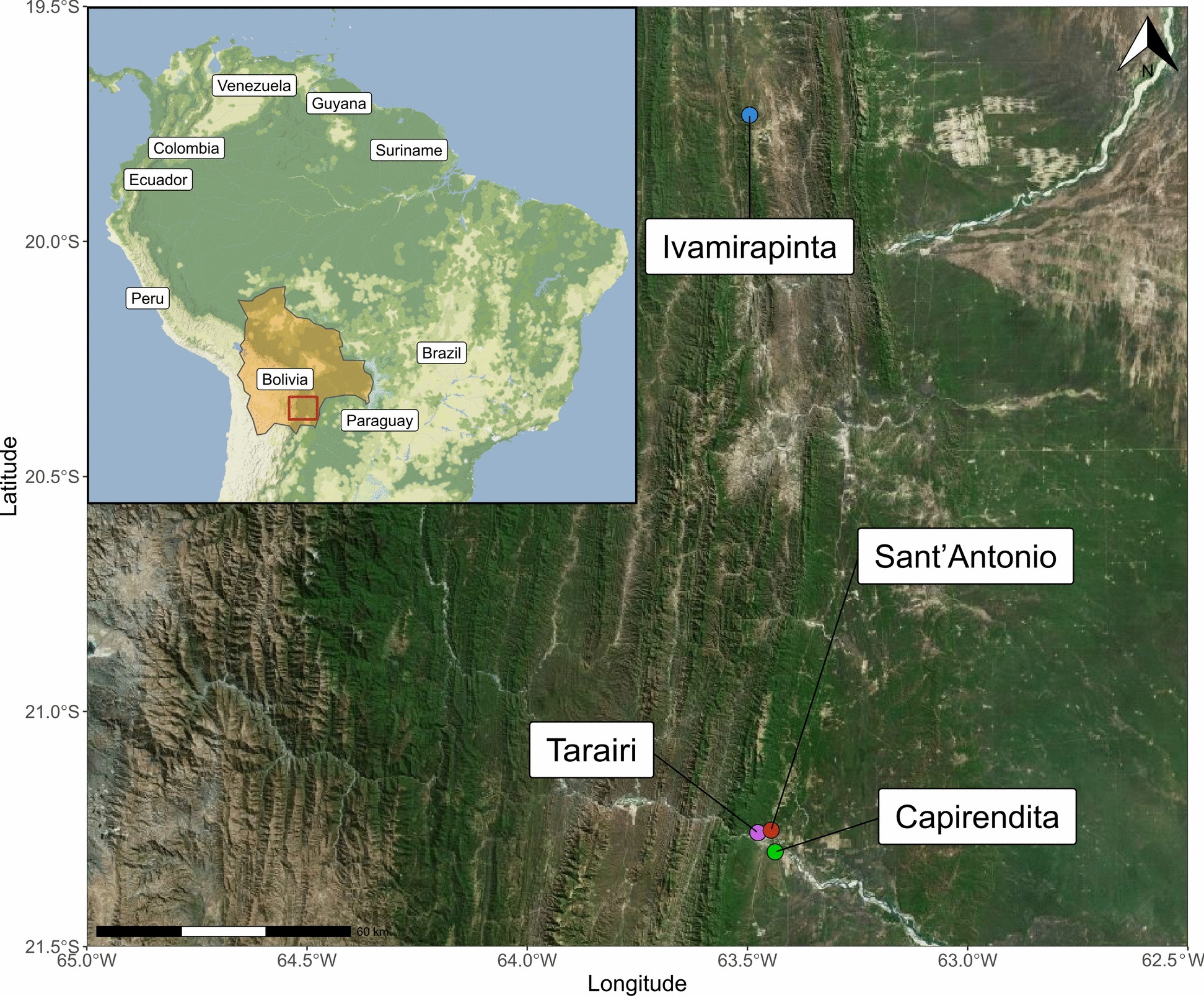
The study area with surveyed communities: Camiri, Department of Santa Cruz (community: Ivamirapinta); Villa Montes, Department of Tarija (communities: Capirendita, Sant’Antonio, Tarairi)

The study was conducted between September and October 2022 in four rural communities located near the small cities of Camiri (Ivamirapinta; Santa Cruz Department) and Villa Montes (Capirendita, Tarairi and Sant’Antonio; Tarija Department) (Fig. 1). Blood samples were collected from both human and canine populations in these locations. Fig. 1 The study area with surveyed communities: Camiri, Department of Santa Cruz (community: Ivamirapinta); Villa Montes, Department of Tarija (communities: Capirendita, Sant’Antonio, Tarairi).

### Sample collection

#### Canine sampling

A total of 189 dogs were enrolled in the study – 95 males and 94 females – from Camiri (n = 46; 29 males and 17 females) and Villa Montes (n = 143; 66 males and 77 females) communities. All animals were mixed-breed and over 12 months of age. Owing to the absence of recent census data, precise calculation of sampling coverage was not possible. Nonetheless, the study aimed to include all accessible owned dogs in the selected villages, using a convenience sampling approach.

Sampling procedures followed the International Guiding Principles for Biomedical Research Involving Animals (Council for International Organizations of Medical Sciences). Dog owners were informed of the study’s objectives, procedures, potential risks and benefits, and they assisted investigators in handling the animals during sampling to minimise the risk of injury.

For each dog, data were recorded on sex, age, breed, living conditions and the presence of clinical signs suggestive of leishmaniasis. Blood samples were collected from the cephalic vein and stored in two separate tubes: one containing anticoagulant (K3EDTA) and the other with a clot activator.

Of the 189 canine samples collected, 125 were subjected to molecular testing for *Leishmania* spp., owing to limitations in sample volume and quality affecting DNA extraction efficiency. Similarly, 164 samples were analysed serologically for *Trypanosoma cruzi*; the remainder were excluded due to sample unavailability or haemolysis. These limitations reduced the total number of samples available for each type of analysis.

#### Human sampling

In October 2022, a parasitological survey was conducted in nine rural communities of the Bolivian Chaco, including the four study sites, as part of a broader monitoring programme for soil-transmitted helminths. A total of 492 school-aged children (SAC) were recruited. In each community, at least 50 children attending the third year of primary school – typically aged 8–10 years – were enrolled.

The selected sample size (*n* = 50 per cluster) is based on WHO guidelines for cluster sampling, assuming a design effect of 2. This approach allows estimation of prevalence with 5% absolute precision at a 95% confidence level, which is deemed sufficient for monitoring in endemic settings (WHO, 2011).

Written informed consent was obtained from parents or legal guardians. Children were provided with age-appropriate explanations to ensure informed assent. The study was conducted with the support of the Bolivian Ministry of Health (Convenio Ministerio de Salud) and the Guaraní political organisation (Asamblea del Pueblo Guaraní) and received ethical approval from the local ethics committee (Colegio Médico de Santa Cruz, TDEM CITE No. 005/2016), in compliance with local and international standards, including the Declaration of Helsinki.

Blood samples were collected from all participants via finger-prick and stored as dried blood spots (DBSs) on Whatman® Grade 3 filter paper. Approximately 25 µL of blood was applied to each preprinted circle (1.2 cm diameter).

### Laboratory procedures

#### Detection of *Leishmania* spp. in dogs and humans

Canine sera were tested for anti-*Leishmania* antibodies using a rapid diagnostic test (RDT) (Cypress Diagnostics, Belgium; sensitivity: 95%, specificity: 100%, per manufacturer) and an indirect ELISA (VET-Innovate ID Diagnostics, France; sensitivity: 85%, specificity: 90%).

Human sera were extracted from DBSs following established protocols [23]. Each spot was eluted in 200 µL of PBS-Tween buffer, resulting in an approximate final dilution of 1:10. ELISA testing was then performed using the Visceral Leishmaniasis kit (Cypress Diagnostics, Belgium; sensitivity: 87%, specificity: 92%).

Genomic DNA was extracted from 200 µL of whole blood (EDTA) using the QiAmp DNA Blood Mini Kit (Qiagen, USA), following the manufacturer’s instructions. DNA from DBSs was eluted overnight in 180 µL ATL buffer at room temperature prior to purification with the same kit.

All extracted DNA samples were tested using a qPCR assay with the Genesig® *Leishmania* All Species Standard Kit (UK; sensitivity: 95%, specificity: 98%). Samples that tested positive by qPCR were further amplified via conventional PCR using the L5.8S/LITSR primers targeting a ~ 300 bp fragment of the ITS1 region [24]. Amplicons were purified and sequenced bidirectionally using the same primers. Resulting sequences were aligned using ClustalW [25] and compared with GenBank entries via BLASTn (http://blast.ncbi.nlm.nih.gov/Blast.cgi).

#### Detection of Trypanosoma cruzi in dogs

Detection of anti-*T*. *cruzi* IgG in canine sera was performed using a rapid diagnostic test (Citest Diagnostics, Canada; sensitivity: 88%, specificity: 92%). Additionally, DNA was tested using a qPCR assay (Genesig® *Trypanosoma cruzi* Standard Kit, UK; sensitivity: 97%, specificity: 99%) in accordance with previously described protocols [22].

### Statistical analysis

Statistical analysis was carried out using STATA version 18.0 BE-Basic Edition (StataCorp, College Station, Texas, USA). Univariate analyses were conducted using chi-squared tests to assess associations between pathogen prevalence and potential risk factors, including sampling site, demographic characteristics, presence of clinical signs and co-infections. A *p*-value of ≤ 0.05 was considered statistically significant.

## Results

A total of 87 (46%, CI 95%: 38.7–53.4%) of the 189 dogs enrolled from the Camiri area (*N* = 46; 29 males and 17 females) and the Villa Montes communities (*N* = 143; 66 males and 77 females) tested positive for *Leishmania* serology (ELISA or rapid immunochromatographic assay). All animals were of mixed breed and aged over 12 months. Among the positive cases, 29.8% were reactive in both serological tests, with a Cohen’s kappa coefficient of 0.69, indicating moderate agreement.

Seroprevalence was significantly higher in Villa Montes (52.4%; 95% CI 43.9–60.8%) compared with Camiri (26.0%; 95% CI 14.2–41.1%) (χ^2^ = 10.03, df = 1, p < 0.001) (Table 1). Clinical signs consistent with CanL were absent in all seropositive dogs from Camiri, whereas 80% of infected dogs in Villa Montes were symptomatic.

**Table 1:** Serological and molecular findings from blood samples collected from dogs

Parasite species	Method	Camiri	Villa Montes	Total
	ELISA	5/46 (10.9%)	46/143 (32.2%)	51/189 (27.0%)
*Leishmania* spp.	RDT	7/46 (15.2%)	53/143 (37.0%)	60/189 (31.7%)
	Total seroprevalence	12/46 (26.0%)	75/143 (52.4%)	87/189 (46.0%)
	qPCR	0/46 (0%)	9/79 (11.4%)	9/125 (7.2%)
*Trypanosoma cruzi*	RDT	6/46 (13.0%)	23/118 (19.5%)	29/164 (17.7%)
	qPCR	0/46 (0%)	0/79 (0%)	0/125 (0%)

The proportion of positive samples for each method is expressed as a percentage of the total samples analysed. Total seroprevalence refers to the number of samples that tested positive for *Leishmania* spp. using either ELISA or a rapid immunochromatographic assay (RDT)

Molecular screening by qPCR, performed on 125 canine blood samples, detected *Leishmania* DNA in 9 dogs (7.2%), with Ct values ranging from 32 to 35 (estimated 20 to 2 copies per reaction; R^2^ = 0.939). All qPCR-positive animals originated from Villa Montes (Table 1). BLAST analysis revealed 98–100% sequence identity with *Leishmania infantum* (GenBank accession no. MW930738.1).

Regarding *Trypanosoma cruzi*, the overall seroprevalence was 17.7% (95% CI 12.1–24.4%) among the 164 dogs tested (Camiri: n = 46; Villa Montes: n = 118). Seroprevalence was 13.0% (95% CI 4.9–26.3%) in Camiri and 19.5% (95% CI 12.7–27.8%) in Villa Montes. None of the dogs tested positive for *T*. *cruzi* DNA by qPCR (Table 1).

No statistically significant differences in infection rates were observed between male and female dogs for either *Leishmania* spp. or *T*. *cruzi* (χ^2^ = 2.11, df = 1, *p* = 0.1448). Notably, 14 of the 29 T*. cruzi*-seropositive dogs (48.3%; 95% CI 29.4–67.5%) were also seropositive for *Leishmania* spp. (Additional file 1: Table S1).

Among the human population, none of the 83 school-aged children (SAC) from Camiri (49 girls, 34 boys; mean age: 9.5 years) tested positive for *Leishmania* antibodies. In contrast, 9 out of 68 children from Villa Montes (31 girls, 37 boys; mean age: 10.2 years) were seropositive, corresponding to a seroprevalence of 13.2% (95% CI 6.2–23.6%). No children displayed clinical signs of leishmaniasis, and all dried blood spot (DBS) samples tested negative by qPCR. No significant differences in *Leishmania* seroprevalence were observed by sex or age among SAC (χ^2^ = 2.11, df = 1, *p* = 0.452).

## Discussion

This study highlights the relevance of adopting a One Health approach to understand the dynamics of *Leishmania infantum* and *Trypanosoma cruzi* infections in the Bolivian Chaco. Dogs play a critical role as reservoir hosts in the zoonotic transmission cycle of *L*. *infantum*, especially in peri-urban areas with close human–animal interactions.

In Villa Montes, a surveillance campaign conducted in September 2022 identified 121 infected dogs among 816 sampled (Zoonosis Unit, Villa Montes, personal data). Within this context, our integrated diagnostic approach – combining two serological assays and a qPCR test – revealed a seroprevalence of 46% among 189 dogs, with 7.2% actively infected as confirmed by *Leishmania* DNA detection.

The moderate agreement between serological assays (κ = 0.69) aligns with previous reports (e.g. κ = 0.47) [28] and reflects their inherent differences in diagnostic performance. Given that seropositivity does not confirm active infection, qPCR was included to improve specificity, although blood samples may underestimate parasite load, particularly in chronic or subclinical cases [29]. Tissue samples (e.g. lymph nodes or bone marrow) are more sensitive but were not feasible owing to ethical and logistical constraints in the field.

All *Leishmania* DNA-positive samples were identified as *L*. *infantum*, consistent with earlier reports from the region. This has significant public health implications, particularly as human seropositivity was also detected in Villa Montes, though no active infections or clinical symptoms were recorded. Serological testing from DBS samples, validated in recent studies [30], provided a non-invasive and field-appropriate alternative, though qPCR sensitivity may have been compromised by low parasitaemia or DNA degradation [30, 31].

The absence of seropositive children in Camiri contrasts with the epidemiological situation in Villa Montes, suggesting differences in transmission intensity, potentially driven by ecological and climatic factors. Vector distribution is influenced by variables such as altitude, temperature and humidity [32, 33]. Camiri, at 812 m a.s.l., experiences cooler and less humid conditions compared with Villa Montes (388 m a.s.l.), which likely favours sand fly proliferation in the latter. Similar altitude-driven patterns have been reported in *Lutzomyia longipalpis* distribution in Colombia [34].

Environmental factors such as open waste disposal and poverty-related conditions in Villa Montes may further support vector breeding. Comparable circumstances in Salta province, Argentina, near the Bolivian border, have shown canine *L*. *infantum* seroprevalence of 13% [35], reinforcing the notion of a cross-border endemic zone.

Regarding *T*. *cruzi*, 17.7% of dogs were seropositive, but none tested positive by qPCR, suggesting chronic infections with low parasitaemia. These results align with previous findings in the Bolivian Chaco [22] and Argentine Chaco [36], where canine seroprevalence varied widely. The high co-seropositivity with *L*. *infantum* (48.3%) raises the possibility of co-infection or serological cross-reactivity. Although cross-reactivity is known between *T*. *cruzi* and *L*. *infantum*, our combined serological and qPCR approach aimed to reduce false positives [37]. Nevertheless, negative PCR results warrant cautious interpretation, particularly in chronic infections or when using peripheral blood samples.

Collectively, our findings emphasize the need for strengthened vector control, surveillance and intersectoral coordination to manage the zoonotic risk posed by both *L*. *infantum* and *T*. *cruzi*. Potential wildlife reservoirs and ecological drivers also merit further investigation [38].

## Conclusions

This study underscores the urgent need to implement integrated One Health strategies to address the burden of vector-borne zoonoses in the Bolivian Chaco. The detection of high *L*. *infantum* seroprevalence in dogs, active infections confirmed by qPCR, and human seropositivity in Villa Montes indicate ongoing transmission and the risk of zoonotic spillover.

Sustainable control strategies should incorporate targeted vector control interventions, canine reservoir management, enhanced diagnostic surveillance and community engagement.

Future research should prioritize spatial mapping of infection hotspots and identification of ecological drivers to better understand transmission dynamics. Cross-disciplinary collaboration among veterinary, medical and environmental sectors will be essential to mitigate the impact of *L*. *infantum* and *T*. *cruzi*, and to strengthen public health resilience in this vulnerable region.

## Supplementary Information

Additional file 1.

## Data Availability

Data supporting the main conclusions of this study are included in the manuscript.
