# Supplementary material for: Neglected parasitic diseases from a one-health perspective: American trypanosomiasis and leishmaniasis in dogs and humans in the Bolivian Chaco
Source: Parasit Vectors. 2025 Nov 27;18:514. doi: 10.1186/s13071-025-07044-y (PMC12752051; doi:10.1186/s13071-025-07044-y)
Supplement: Supplementary file 1 — Additional file 1. [file 13071_2025_7044_MOESM1_ESM.docx]

| **Sample ID** | **Results** | | | | |
| --- | --- | --- | --- | --- | --- |
|  | **Ls qPCR** | **Ls ELISA** | **Ls RDT** | **Tc RDT** | **Tc qPCR** |
| Ca13 | - | + | - | + | - |
| Ca20 | - | - | + | + | - |
| VM62 | + | - | + | + | - |
| VM77 | - | - | + | + | - |
| VM86 | - | - | + | + | - |
| VM105 | - | - | + | + | - |
| VM108 | - | - | + | + | - |
| VM113 | - | - | + | + | - |
| VM118 | + | + | + | + | - |
| VM120 | - | + | + | + | - |
| VM128 | - | + | + | + | - |
| VM130 | - | + | + | + | - |
| VM135 | - | + | + | + | - |
| VM138 | - | + | - | + | - |

Table S1: List of the dogs tested positive for *Trypanosma cruzi* and *Leishmania* spp. using qPCR, ELISA and Rapid Diagnostic Test (RDT), enrolled in the Camiri (Ca) and Villa Montes (VM) areas.Inizio modulo
